# Universal Temporal Profile of Replication Origin Activation in Eukaryotes

**DOI:** 10.1371/journal.pone.0005899

**Published:** 2009-06-12

**Authors:** Arach Goldar, Marie-Claude Marsolier-Kergoat, Olivier Hyrien

**Affiliations:** 1 Commissariat à l'Energie Atomique (CEA), iBiTec-S, Gif-sur-Yvette, France; 2 Ecole Normale Supérieure, UMR CNRS 8541, Paris, France; University College London, United Kingdom

## Abstract

Although replication proteins are conserved among eukaryotes, the sequence requirements for replication initiation differ between species. In all species, however, replication origins fire asynchronously throughout S phase. The temporal program of origin firing is reproducible in cell populations but largely probabilistic at the single-cell level. The mechanisms and the significance of this program are unclear. Replication timing has been correlated with gene activity in metazoans but not in yeast. One potential role for a temporal regulation of origin firing is to minimize fluctuations in replication end time and avoid persistence of unreplicated DNA in mitosis. Here, we have extracted the population-averaged temporal profiles of replication initiation rates for *S. cerevisiae*, *S. pombe*, *D. melanogaster*, *X. laevis* and *H. sapiens* from genome-wide replication timing and DNA combing data. All the profiles have a strikingly similar shape, increasing during the first half of S phase then decreasing before its end. A previously proposed minimal model of stochastic initiation modulated by accumulation of a recyclable, limiting replication-fork factor and fork-promoted initiation of new origins, quantitatively described the observed profiles without requiring new implementations.

The selective pressure for timely completion of genome replication and optimal usage of replication proteins that must be imported into the cell nucleus can explain the generic shape of the profiles. We have identified a universal behavior of eukaryotic replication initiation that transcends the mechanisms of origin specification. The population-averaged efficiency of replication origin usage changes during S phase in a strikingly similar manner in a highly diverse set of eukaryotes. The quantitative model previously proposed for origin activation in *X. laevis* can be generalized to explain this evolutionary conservation.

## Introduction

Eukaryotic chromosome replication starts at multiple positions called replication origins [1], [2]. Origins are activated at different times through S phase. DNA synthesis progresses bidirectionally from each origin, and stops when replication forks converge. Although examination of cell populations suggests that chromosomes replicate according to a precise spatiotemporal program [3]–[7], analysis of individual replicating chromosomes show that this program is largely probabilistic as no two cells replicate their chromosomes in exactly the same manner [8], [9]. Origins in a broad range of eukaryotes show broadly distributed efficiencies and firing times [2], [10], [11].

Why a replication program exists at all is not clear. In metazoans, early replication has been correlated with gene transcription, and replication timing has been proposed to facilitate the propagation of alternative epigenetic chromatin states during chromosome duplication [12]. However, early replication is not correlated with transcription in budding yeast [3]. Another proposed role for a regulated timing of origin activation is to ensure a reproducible replication end time despite the stochasticity discussed above. Stochastic initiation implies fluctuations in replication end time and occasional persistence of unreplicated DNA in mitosis, with ensuing inviability of daughter cells [13], [14]. The distribution of such fluctuations depends, among other parameters, on the time-dependent rate of origin usage [15], [16].

This problem has been much studied in early Xenopus embryos because their origins are placed randomly and initiate stochastically [14], [17]–[20]. To explain how stochastic initiation can result in a reliable replication completion time, it was proposed that potential origins are redundant and that the time-dependent rate of initiation (number of initiations per time unit per unit length of unreplicated DNA), *I(t)*, increases during S phase to speed up replication of unreplicated DNA stretches [14], [19]. Early DNA combing measurements indeed suggested that *I(t)* increases throughout S phase [21]–[24]. As fork progression is approximately constant [17], [18], [20], [25], *I(t)* entirely determines the distribution of replication end time and the maximal number of forks that will assemble on chromatin. Mathematical analysis has shown that given a maximal allowable fork density, an increasing *I(t)* minimizes the risk of incomplete replication by allowing fork proteins to be efficiently reused [15], [16].

**Figure 1:**
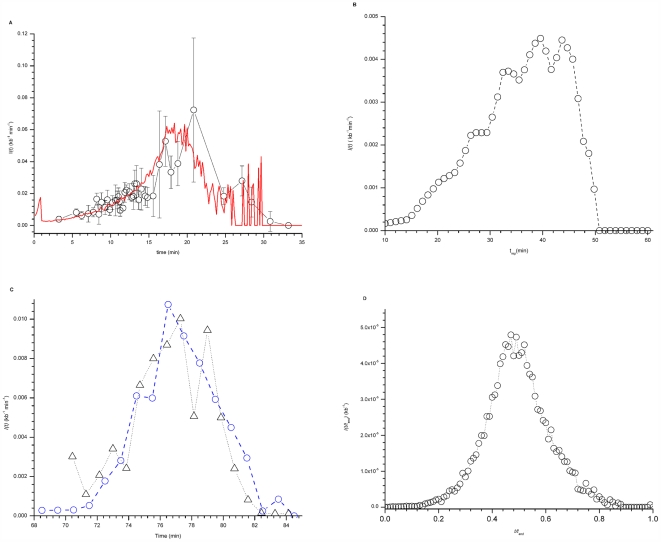
*I(t)* in *X. laevis*, *S. cerevisiae*, *S. pombe* and *D. melanogaster*. A. *I(t)* in *X. laevis*. *I(t)* was determined from DNA combing data (open circles) as described in Materials and Methods in [26]. The data points were binned into 40 points and the dispersion in data value of each bin was determined (gray standard deviation bars). The red line is the best fit of the numerical model [26] to the data (χ^2^ = 0.89). The values of free parameters as described in [26] are: P_0_ = 1.05×10^−4^±1.07×10^−7^ kb^−1^ s^−1^, P_1_ = 2.1×10^−3^±2×10^−5^ kb^−1^ s^−1^, J = 5±0.5 s^−1^ and N_0_ = 1000±3. B. *I(t)* in *S. cerevisiae* was determined from the replication timing profiles of Raghuraman et al. [3] as described in the Materials and Methods. C. *I(t)* in *S. pombe*. Only the 401 strong origins defined by Heichinger et al. [4] (blue circles) and 516 potential origins defined by Eshaghi et al. [5] (black triangles) were considered. D. *I(t/t_end_)* for *D. melanogaster* determined from replication timing profile of chromosome 2L [28].

More recent data [26], however, have shown that *I(t)* increases until mid S phase but then sharply decreases before the end of S phase (Figure 1A). This behavior could be quantitatively explained by a minimal stochastic model in which the propensity of origins to fire depends on both the gradual accumulation of a limiting, recyclable replication-fork factor and a fork-promoted initiation of new origins [26]. The model strictly required the combination of both these ingredients to work.

To determine whether the initiation function that evolution has selected in early *Xenopus* embryos reflects general constraints or a peculiarity of this system, we decided to extract the *I(t)* in *S. cerevisiae*, *S. pombe*, *D. melanogaster* and *H. sapiens* from published genome-wide replication timing profiles (as detailed in the Materials and Methods) and to compare them to *X. laevis*, with the idea that if comparable *I(t)* functions were found, the model developed for *Xenopus* embryos could more generally apply.

## Results

The time-dependent rate of replication initiation *I(t)* in the budding yeast *S. cerevisiae* was constructed using the genome-wide replication profiles determined by Raghuraman et al [3], where the mean replication time of each studied locus is plotted against chromosomal position so that efficient origins appear as peaks. The replication profile of each chromosome was cut into one-minute slices of time. *I(t)* was calculated for each time point as the ratio between the number of fired origins and the amount of unreplicated DNA. Figure 1B shows that the rate of initiation increases during the first 35–40 min of S phase then decreases sharply before the end of S phase (60 min), as observed in *Xenopus* egg extracts [24], [26] (Fig. 1A). Since both DNA combing and microarray data are population averages, it should be noted that the *I(t)* extracted from all the organisms discussed here are population averages.

We similarly calculated *I(t)* in the fission yeast *S. pombe*, using the independent microarray studies of Heichinger et al. [4] and Eshaghi et al. [5], who studied synchronous cells released from a block in late G2 or with hydroxyurea (HU), respectively. Heichinger et al. [4] calculated a minimum length of S-phase of 19 min. Eshaghi et al. [5] found that the duration of S phase after HU release was ∼60 min. It has been shown that in *S. cerevisiae* the pattern of origin activation is not affected by continuous exposure to HU, although HU causes an expansion of S phase time frame [27]. Assuming this is also the case in *S. pombe*, we have divided the S phase time frame of Eshaghi et al. by a factor of 3 and translated it by 70 min so as to match the S phase start and end times of Heichinger et al. Figure 1C shows that when the data are plotted this way, the time-dependent rates of origin firing determined from both experiments indeed coincide. Again, *I(t)* increases during the first half of S phase then decreases sharply before the end of S phase, as observed in *Xenopus*.

To similarly calculate *I(t)* in *D. melanogaster*, we used the replication timing profile of chromosome 2L obtained by MacAlpine et al [28] and a peak-finding algorithm to detect initiation events. Because the absolute length of S phase was not specified in this study, we plotted the extracted rate of initiation as a function of the relative time in S phase, (*t/t*_end_), where *t_end_* is the time at which DNA is fully replicated. As shown in Figure 1D, the rate of origin initiation increases during the first half of S phase then decreases before the end of S phase.

**Figure 2:**
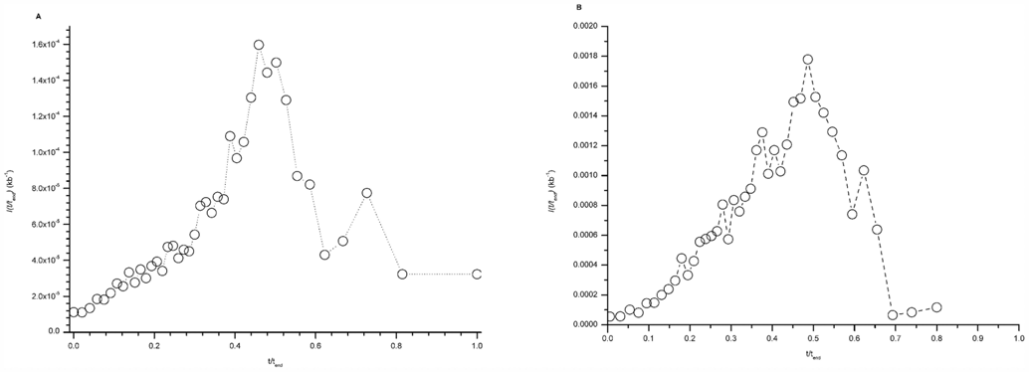
*I(t)* in *Homo sapiens*. *I(t)* was determined from the replication timing profiles determined by Woodfine et al. [6], [7] for: (A) the whole genome, at 1 Mb resolution [7] or (B) chromosome 6, at 94 kb resolution [6], as described in the Materials and Methods.

To calculate *I(t)* in *H. sapiens*, we used the genome-wide and chromosome 6 maps of replication timing in human lymphoblastoid cells obtained by Woodfine et al [6], [7]. In contrast to the yeast genome-wide studies, the resolutions of these two studies were insufficient to precisely determine the position of replication origins. However, peaks in the replication timing profiles must contain at least one origin, so that a minimal number of initiation events at different time points throughout S phase could be extracted from the replication profiles. The calculated rates of initiation *I*(*t/t*_end_) are shown on Figure 2A and 2B. The profiles of *I*(*t/t*_end_), obtained using either the genome-wide data (Figure 2A) or the chromosome 6 data (Figure 2B), have very similar shapes although the maximal amplitude of *I*(*t/t*_end_) was higher for the better-resolved chromosome 6 data than for the genome wide data by a factor of 10. The chromosome 6 data were obtained using a 94 kb resolution tiling path array, whereas the genome wide data were obtained using an array of probes (mean size 150 kb) spaced at 1 Mb intervals. Therefore, a higher proportion of origins was detected using the chromosome 6 data. The fact that the shape of *I*(*t/t*_end_) is not altered when resolution is changed by a factor of 10, suggests that a similar fraction of initiation events are detected throughout S phase when sampling is suboptimal. Once again, the rate of origin activation increases during the first half of S phase then decreases sharply before the end of S phase.

**Figure 3:**
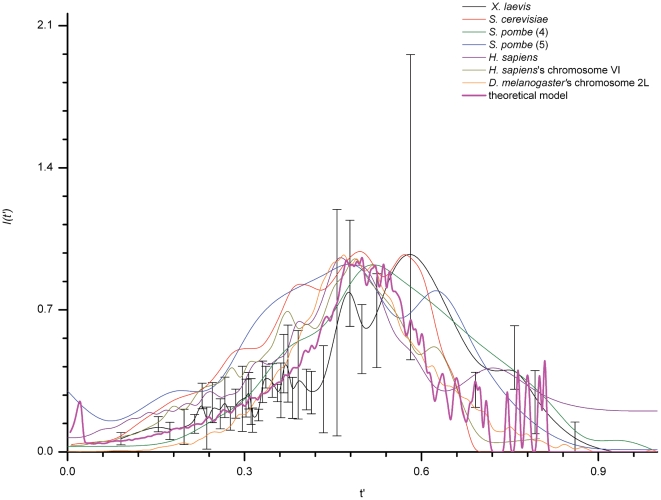
Collapse of all *I(t')*. All curves were shifted horizontally so that their starting points coincide with zero. The similarity distance (d_sim_) between the *I(t')* of other eukaryotes and the *I(t')* of *X. laevis* (Black) was measured using the Continuous Dynamic Time Warping method (see Material and Methods). By using the *X. laevis* data dispersion (gray error bars) we set the condition that if the measured distance between two curves is smaller than 0.94, the two curves are similar. In all cases we found d_sim_<0.94, therefore all *I(t')*, including the one generated by the numerical model, could be considered as similar. However, its possible to define a sequence of decreasing similarity between considered *I(t')* as: *H. sapiens* ‘s chromosome 6 (d_sim_ = 0.27, Dark yellow)>*D. melanogaster* (d_sim_ = 0.38, Orange)>numerical model (d_sim_ = 0.41, Magenta)>*S. pombe* from Heichinger et al (d_sim_ = 0.43, Olive)>*H. sapiens* (d_sim_ = 0.44, Purple)>*S. pombe* from Eshaghi et al (d_sim_ = 0.55, Blue)>*S. cerevisiae* (d_sim_ = 0.85, Red).

The time-dependent rate of replication origin activation, *I(t)*, is defined as an average over the whole genome. Therefore it is independent of chromosomal position and represents the population-averaged dynamic process that controls origin usage and replication fork density, whether calculated from DNA combing or replication timing profiles. The theoretical *I(t)* calculated using the previously developed stochastic model is also an average of 500 simulations. The *I(t)* calculated either way can thus be compared. To compare the *I(t)* of different organisms, plots had to be normalized. S phase length and initiation frequency depend on many parameters that can vary among organisms and according to growth conditions. For example HU-treated *S. cerevisiae* cells exhibit an extended S phase compared to untreated cells although their rate of origin initiation is unaffected when related to the fraction of replicated DNA [27]. We therefore normalized replication time with respect to S phase length. Similarly, the maximum value of *I(t)* depends on the concentration of factors limiting origin efficiency [29]–[31]. Each *I(t)* was thus normalized by its maximum value and plotted as a function of reduced time *t'* (*t'* = *t/t_end_*). As shown in Figure 3, all the *I(t')* collapse together and have a maximum value at mid S-phase. The calculated agreement with the *X. laevis* data (see legend to Fig 3) appears particularly good for *S. pombe*, which relies on a largely stochastic mechanism for origin initiation, and for *H. sapiens* and *D. melanogaster*. The curve appears broader for *S. cerevisiae*, perhaps because cell synchrony was less tight, or because there is a clearer demarcation of early and late replicating domains in this organism [32]. Importantly, the *I(t')* calculated using the minimal stochastic model that we have previously developed [26] could account for all these observations.

## Discussion

In this work, we have extracted the temporal profiles of replication initiation rates for *S. cerevisiae*, *S. pombe*, *D. melanogaster*, *X. laevis* and *H. sapiens* and have shown that all the profiles have strikingly similar shapes, increasing during the first half of S phase then decreasing before its end. A minimal model of stochastic initiation modulated by accumulation of a recyclable, limiting replication-fork factor and fork-promoted initiation of new origins, quantitatively describes the observed profiles.

It should be noted that several factors could affect the shape of the extracted *I(t)* profiles. As replication timing maps are population averages, assigning an initiation to a particular time (the midpoint of the probability distribution of initiations for that origin) is an approximation. Depending on the shape of each origin's firing time distribution, the changes in *I(t)* at the start and the end of S phase may not be exactly as portrayed since initiations can occur before or after the assigned timepoint of the origin in the data set. There are not enough data on initiation time probability distributions to take this factor into account in our *I(t)* estimates. We therefore assume that population-averaged *I(t)* is a reasonable enough estimate of *I(t)* at the single cell level, although more work would be required to conclude on this point. More importantly, our approach only counts origins that are efficient enough to trace a peak in the timing profiles. Origin efficiency may change during S phase, which could affect the *I(t)* profile. The efficiency of origin usage has been estimated by two different methodologies in two independent studies in *S. pombe*. Heichinger et al. [4] concluded that early origins tend to be more efficient than late origins whereas Eshagi et al. [5] concluded the opposite. As the *I(t)* profiles extracted from both studies are strikingly similar, we tend to think that changes in origin efficiency are not large enough to significantly affect our conclusions.

The organisms studied here include two extreme systems of eukaryotic DNA replication. Replication in *Xenopus* early embryos is largely stochastic since initiation is mostly random in space and time [14], [33]. In contrast, initiation in *S. cerevisiae* is submitted to stronger spatial and temporal constraints, having efficient, site-specific origins, whose activation times appear fairly fixed [3], [32]. Other organisms exhibit an intermediate behavior [2]. However, all eukaryotes including *S. cerevisiae* present an excess of potential origins compared to the ones actually used during a given S phase [1] and this redundancy confers randomness even to sequence-determined origin locations [9]. The similarity of *I(t')* across all the data sets analyzed suggests that even though the molecular details of the initiation process and the degree of stochasticity in origin activation differ, all these organisms exhibit the same dynamic behavior of origin firing. The scheme that emerges from the collective action of numerous replication factors is the same and bypasses the complexity of each network; the behavior of *I(t)* is universal over such details.

What are the mechanisms and evolutionary significance of the universal shape of *I(t)*? Our model contains the minimal ingredients required to account for the shape of *I(t)* in a quantitative manner [26]. These ingredients include origin redundancy, stochasticity of firing times, a recyclable, limiting factor whose concentration increases during S phase, and a dependence of origin activation on fork density. A universal feature of eukaryotic cells is the confinement of their genome in the cell nucleus and the consequent need to import replication factors in the nucleus during S phase [34]. We have shown that the increase in *I(t)* can be explained by nuclear import of a limiting, recyclable replication protein [26]. An *I(t)* that increases throughout S phase helps to minimize the maximal density of forks required for timely completion of S phase [15], [16]. It remains to be seen how much this holds true when *I(t)* decreases at the end of S phase. We have shown that this decrease can be explained by a self-limiting correlation between fork density and further initiation [26]. Cdc45, a stable fork component, can recruit Cdk2, a kinase involved in origin activation, at replication foci in mammalian cells [35]. Such an interaction may explain the correlation between fork density and origin firing [26], although it is unknown if it is conserved in yeasts and fly. Reducing initiation late in S phase avoids local buildup of forks and simplifies the topological and organizational constraints associated with sister chromatid maturation and resolution. Natural selection might have favored the observed shape of *I(t)* because it is the best compromise between efficient recycling of replication proteins and prevention of excessive fork densities late in S phase.

It is conceivable that other mechanisms than proposed in our model could account for the data equally well [36]–[38]. However, this model appears to contain the minimal number of ingredients sufficient to describe the unique behavior of *I(t)* in a quantitative manner [26], whether replication initiates at specific or at random sequences.

## Materials and Methods

### Calculation of replication initiation rate, *I(t)*, from published replication timing profiles

#### *S. cerevisiae*

The time-dependent rate of replication initiation, *I(t)* in the budding yeast *S. cerevisiae* was constructed using the experimental data of Raghuraman et al [3]. In this study, yeast cells grown with heavy isotope DNA precursors were arrested at the G1/S transition, resuspended in isotopically light medium and allowed to proceed into S phase. DNA samples were taken through S phase, and the replicated (heavy-light, HL) and unreplicated (heavy-heavy, HH) DNA fragments were purified and separately hybridised to arrays carrying probes to the entire genome. For each locus on the array, a %HL_(total)_, which is a direct reflection of the replication time, was calculated and plotted as a function of chromosome coordinate. Peaks represent sites of earliest local replication and must contain a replication origin. Using this method the location and time of activation of the 332 most efficient origins were determined.

To extract the rate of firing of replication origins per unit time and per unit length of unreplicated DNA, *I(t)*, the replication profile of each chromosome was cut into one-minute slices and the extent of chromosome replication at each time was determined. The total fraction of unreplicated DNA was calculated as:
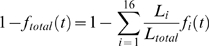
(1)where i ∈ [1], [16] represents the index of each chromosome, *f*_i_ *(t)* the fraction of replicated DNA of each chromosome, *L*_i_ the probed length of each chromosome and *L*_total_ the total probed size of the genome (*L*_total_ = 11982 kb). The firing times of the 332 origins were distributed among 13 bins of 3 min width each. To calculate *I(t)*, the total fraction of unreplicated DNA and the time distribution of origin firing were interpolated over 50 points in the interval of [10,60] minutes using a spline interpolation algorithm. Then for each time point *I(t)* was calculated as:
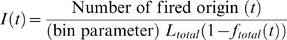
(2)where “bin parameter” is equal to 3 min.

#### *S. pombe*

To calculate the rate of replication origin firing in the fission yeast *S. pombe* we used two independent microarray studies performed by Heichinger et al [4] and by Eshaghi et al [5]. In the first study [4], cells released from a late G2 block were allowed to synchronously progress through S-phase. DNA samples were taken through S phase and hybridised to microarrays covering the coding and noncoding regions of the genome, using DNA from G2-arrested cells as a reference. The increase in DNA content was monitored and the time point at which each segment became 50% replicated was identified and plotted along the chromosomes as a moving average across five probes. In some experiments, hydroxyurea (HU) was added to slow down replication forks and confine DNA synthesis to the vicinity of origins. By combining data from both experiments a total of 401 origins were identified. In the second study [5], cells were released following a hydroxyurea (HU) block. DNA samples taken through S phase hybridized to ORF-specific microarrays. The half-replication time of each locus was plotted along its chromosomal position. Peaks in the profile identified 516 potential origins.

For each data set, the total fraction of unreplicated DNA (1-*f*_total_ *(t)*) was obtained by fitting a Boltzman-sigmoidal function to the temporal changes in the relative DNA content (data points shown in Figure 2A, right panel, in [4] and in Figure 1D in [5]) using a Levenberg-Marquardt algorithm. The distributions of origin firing time were obtained by binning the data in 3 min and 1 min bins, respectively. By using Equation (2) and the *S. pombe* genome size *L*_total_ = 14 Mb, we calculated the rate of replication origin firing, *I(t)*, for each data set.

#### *D. melanogaster*

To calculate the rate of replication origin firing of *D. melanogaster's* chromosome 2L we used high-resolution genomic microarray data obtained by MacAlpine et al. [28]. Kc cells released from a HU-block were pulsed with BrdU at the beginning or end of S phase to specifically label early and late-replicating sequences. BrdU-containing DNA fragments were enriched by immunoprecipitation and differentially labelled and hybridized to a genomic microarray. Replication timing was determined as the ratio of early-to-late BrdU incorporating sequences. Again peaks in the replication profile determine the position of replication origins. The relative replication times and replication fractions were extracted from the early-to-late ratio using 
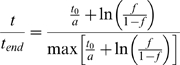
, where 
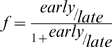
 is the fraction of replicated DNA. To count the number of origins, the positions of peaks were identified by detecting the downward zero-crossings in the first derivative of the smoothed (adjacent averaging over a window that represents 5% of the total data points) early-to-late ratio profiles. If the amplitude of the detected peak exceeded a threshold value (0.01) of the ratio of early to late BrdU incorporation, then the peak was counted as an origin; if not it was discarded. The distribution of the origin firing times was obtained by binning the data in 122 bins of equal width. We plotted the rate of initiation as a function of *t/t_end_*, where *t_end_* is the time at which the DNA is fully replicated (*f_t_*_otal_ = 1). By using Equation (2) and setting the length of chromosome 2L to *L*_total_ = 22.2×10^3^ kb we calculated the rate of initiation *I*(*t/t*_end_).

#### *H. sapiens*

To construct the rate of replication origin firing in human cells, we used the genome-wide and chromosome 6 maps of replication timing in a human lymphoblastoid cell line obtained by Woodfine et al [6], [7]. Nuclei from unsynchronised cells were sorted into S and G1 phases, and the S and G1 DNAs were differentially labelled and simultaneously hybridized to either a genomic clone array probes (mean size 150 kb) spaced at 1 Mb intervals [7] or a chromosome 6 tile path array of BAC and PAC clones whose midpoints were spaced at 94 kb intervals on average [6]. The ratio of S∶G1 DNA (replication timing ratio), was calculated for each clone to estimate the average sequence copy number in the S phase fraction. Values close to 2 indicate an early replicating sequence and values close to 1 a late replicating sequence. Peaks in the profile once again must contain at least one origin, so that a minimal number of initiation events at different time points through S phase could be extracted from the replication profile by counting the number of peaks using the algorithm described above.

To calculate the fraction of replicated DNA, the S∶G1 ratios were distributed over 62 bins of equal width (excluding sex chromosomes, which replicate late in this cell line). The relation between the ratio S∶G1 and the total fraction of the replicated DNA, *f*_total_, is given by: 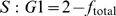. Assuming that the fraction of replicated DNA increases with time in a sigmoidal manner [7], we used a Boltzmann sigmoidal function to estimate the replication time: 
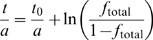
. Because the parameters *t*_0_ and *a* are unknown, we cannot extract the absolute time of replication. Therefore we plotted the rate of initiation as a function of as t/t_end_, where t_end_ is the time at which the DNA is fully replicated (*f_t_*_otal_ = 1). Therefore, by using Equation (2) and setting the total length of the genome to *L*_total_ = 3100×10^3^ kb we could calculate the rate of initiation *I*(*t/t*_end_).

### Calculation of similarity between *I(t')* profiles

To measure the similarity between two calculated *I(t')* we used the continuous dynamic time warping algorithm [39]. In this computational method all possible mappings between two curves are calculated. The similarity between two continuous curves is measured as the shortest path in parameter space in order to map one curve to the other. Therefore, using the dispersion on the *X. laevis* data set we calculated the upper bound limit in similarity distance, d_sim_, below which two curves can be considered similar. We map all *I(t')* curves to *X. laevis* data set and calculate the similarity distance between them.

## References

[1] Gilbert DM (2004). In search of the holy replicator.. Nat Rev Mol Cell Biol.

[2] Hamlin JL, Mesner LD, Lar O, Torres R, Chodaparambil SV (2008). A revisionist replicon model for higher eukaryotic genomes.. J Cell Biochem.

[3] Raghuraman MK, Winzeler EA, Collingwood D, Hunt S, Wodicka L (2001). Replication dynamics of the yeast genome.. Science.

[4] Heichinger C, Penkett CJ, Bahler J, Nurse P (2006). Genome-wide characterization of fission yeast DNA replication origins.. Embo J.

[5] Eshaghi M, Karuturi RK, Li J, Chu Z, Liu ET (2007). Global profiling of DNA replication timing and efficiency reveals that efficient replication/firing occurs late during S-phase in S. pombe.. PLoS ONE.

[6] Woodfine K, Beare DM, Ichimura K, Debernardi S, Mungall AJ (2005). Replication timing of human chromosome 6.. Cell Cycle.

[7] Woodfine K, Fiegler H, Beare DM, Collins JE, McCann OT (2004). Replication timing of the human genome.. Hum Mol Genet.

[8] Patel PK, Arcangioli B, Baker SP, Bensimon A, Rhind N (2006). DNA replication origins fire stochastically in fission yeast.. Mol Biol Cell.

[9] Czajkowsky DM, Liu J, Hamlin JL, Shao Z (2008). DNA combing reveals intrinsic temporal disorder in the replication of yeast chromosome VI.. J Mol Biol.

[10] Dai J, Chuang RY, Kelly TJ (2005). DNA replication origins in the Schizosaccharomyces pombe genome.. Proc Natl Acad Sci U S A.

[11] Rhind N (2006). DNA replication timing: random thoughts about origin firing.. Nat Cell Biol.

[12] Schwaiger M, Schubeler D (2006). A question of timing: emerging links between transcription and replication.. Curr Opin Genet Dev.

[13] Laskey RA (1985). Chromosome replication in early development of Xenopus laevis.. J Embryol Exp Morphol.

[14] Hyrien O, Marheineke K, Goldar A (2003). Paradoxes of eukaryotic DNA replication: MCM proteins and the random completion problem.. BioEssays.

[15] Bechhoefer J, Marshall B (2007). How Xenopus laevis replicates DNA reliably even though its origins of replication are located and initiated stochastically.. Phys Rev Lett.

[16] Yang SC, Bechhoefer J (2008). How Xenopus laevis embryos replicate reliably: Investigating the random-completion problem.. Phys Rev E Stat Nonlin Soft Matter Phys.

[17] Hyrien O, Méchali M (1992). Plasmid replication in Xenopus eggs and egg extracts: a 2D gel electrophoretic analysis.. Nucleic Acids Res.

[18] Hyrien O, Méchali M (1993). Chromosomal replication initiates and terminates at random sequences but at regular intervals in the ribosomal DNA of Xenopus early embryos.. Embo J.

[19] Lucas I, Chevrier-Miller M, Sogo JM, Hyrien O (2000). Mechanisms Ensuring Rapid and Complete DNA Replication Despite Random Initiation in Xenopus Early Embryos.. J Mol Biol.

[20] Mahbubani HM, Paull T, Elder JK, Blow JJ (1992). DNA replication initiates at multiple sites on plasmid DNA in Xenopus egg extracts.. Nucleic Acids Res.

[21] Herrick J, Jun S, Bechhoefer J, Bensimon A (2002). Kinetic model of DNA replication in eukaryotic organisms.. J Mol Biol.

[22] Herrick J, Stanislawski P, Hyrien O, Bensimon A (2000). Replication Fork Density Increases During DNA Synthesis in X. laevis Egg Extracts.. J Mol Biol.

[23] Marheineke K, Hyrien O (2001). Aphidicolin triggers a block to replication origin firing in Xenopus egg extracts.. J Biol Chem.

[24] Zhang H, Bechhoefer J (2006). Reconstructing DNA replication kinetics from small DNA fragments.. Phys Rev E Stat Nonlin Soft Matter Phys.

[25] Marheineke K, Hyrien O (2004). Control of replication origin density and firing time in Xenopus egg extracts: role of a caffeine-sensitive, ATR-dependent checkpoint.. J Biol Chem.

[26] Goldar A, Labit H, Marheineke K, Hyrien O (2008). A dynamic stochastic model for DNA replication initiation in early embryos.. PLoS ONE.

[27] Alvino GM, Collingwood D, Murphy JM, Delrow J, Brewer BJ (2007). Replication in hydroxyurea: it's a matter of time.. Mol Cell Biol.

[28] MacAlpine DM, Rodriguez HK, Bell SP (2004). Coordination of replication and transcription along a Drosophila chromosome.. Genes Dev.

[29] Patel PK, Kommajosyula N, Rosebrock A, Bensimon A, Leatherwood J (2008). The Hsk1/Cdc7 Replication Kinase Regulates Origin Efficiency.. Mol Biol Cell.

[30] Krasinska L, Besnard E, Cot E, Dohet C, Mechali M (2008). Cdk1 and Cdk2 activity levels determine the efficiency of replication origin firing in Xenopus.. Embo J.

[31] Nougarede R, Della Seta F, Zarzov P, Schwob E (2000). Hierarchy of S-phase-promoting factors: yeast Dbf4-Cdc7 kinase requires prior S-phase cyclin-dependent kinase activation.. Mol Cell Biol.

[32] McCune HJ, Danielson LS, Alvino GM, Collingwood D, Delrow JJ (2008). The Temporal Program of Chromosome Replication: Genome-wide Replication in clb5{Delta} Saccharomyces cerevisiae.. Genetics.

[33] Labit H, Perewoska I, Germe T, Hyrien O, Marheineke K (2008). DNA replication timing is deterministic at the level of chromosomal domains but stochastic at the level of replicons in Xenopus egg extracts.. Nucleic Acids Res.

[34] Walter J, Sun L, Newport J (1998). Regulated chromosomal DNA replication in the absence of a nucleus.. Mol Cell.

[35] Alexandrow MG, Hamlin JL (2005). Chromatin decondensation in S-phase involves recruitment of Cdk2 by Cdc45 and histone H1 phosphorylation.. J Cell Biol.

[36] Gauthier MG, Bechhoefer J (2009). Control of DNA replication by anomalous reaction-diffusion kinetics.. Phys Rev Lett in press.

[37] Lygeros J, Koutroumpas K, Dimopoulos S, Legouras I, Kouretas P (2008). Stochastic hybrid modeling of DNA replication across a complete genome.. Proc Natl Acad Sci U S A.

[38] Spiesser TW, Klipp E, Barberis M (2009). A model for the spatiotemporal organization of DNA replication in Saccharomyces cerevisiae.. Mol Genet Genomics.

[39] Efrat A, Fan QF, Venkatasubramanian S (2007). Curve matching, time warping, and light fields: New algorithms for computing similarity between curves.. J Math Imaging Vis.

